# Pathogenic Characteristics of Shrimp Early Mortality Syndrome (EMS)‐Causing *Vibrio parahaemolyticus*: A Comparative Transcriptomic Study Suggests the Relationship Between Metabolic Fitness and Virulence Gene Expression

**DOI:** 10.1111/1758-2229.70219

**Published:** 2025-11-25

**Authors:** Nalumon Thadtapong, Varodom Charoensawan, Vanvimon Saksmerprome, Soraya Chaturongakul

**Affiliations:** ^1^ National Institute of Health, Department of Medical Sciences Ministry of Public Health Nonthaburi Thailand; ^2^ Department of Biochemistry, Faculty of Science Mahidol University Bangkok Thailand; ^3^ Integrative Computational BioScience (ICBS) Center Mahidol University Nakhon Pathom Thailand; ^4^ Division of Medical Bioinformatics, Research Department, Faculty of Medicine Siriraj Hospital Mahidol University Bangkok Thailand; ^5^ Department of Biochemistry, Faculty of Medicine Siriraj Hospital Mahidol University Bangkok Thailand; ^6^ Siriraj Genomics, Faculty of Medicine Siriraj Hospital Mahidol University Bangkok Thailand; ^7^ School of Chemistry, Institute of Science Suranaree University of Technology Nakhon Ratchasima Thailand; ^8^ Center of Excellence for Shrimp Molecular Biology and Biotechnology, Faculty of Science Mahidol University Bangkok Thailand; ^9^ National Center for Genetic Engineering and Biotechnology (BIOTEC) Pathumthani Thailand; ^10^ Center for Advanced Therapeutics, Institute of Molecular Biosciences Mahidol University Nakhon Pathom Thailand; ^11^ Pornchai Matangkasombut Center for Microbial Genomics (CENMIG), Faculty of Science Mahidol University Bangkok Thailand

**Keywords:** AHPND, early mortality syndrome, non‐AHPND, transcriptomics, *V. parahaemolyticus*

## Abstract

*Vibrio parahaemolyticus* (VP) is a major bacterial species that causes early mortality syndrome (EMS) in shrimps. EMS can be classified into two groups based on histological signs of hepatopancreatic tissues, i.e., acute hepatopancreatic necrosis disease (AHPND) and non‐AHPND. To investigate how toxin‐producing AHPND and toxin‐lacking non‐AHPND VP could lead to EMS, growth characteristics and transcriptomic analyses of the representative strains, 5HP and 2HP, were compared. Non‐pathogenic VP represented by strain S02 was also used. Two types of growth media included rich medium represented by tryptic soy broth plus 1.5% NaCl (TSB) and depleted media represented by artificial seawater (SW) and spent seawater (SSW). SSW refers to a sterile used‐SW medium from healthy shrimp rearing or shrimp‐conditioned SW. Growth characteristics under these media indicated that TSB and SSW supported better growth than SW, suggesting shrimp‐conditioned SW is sufficient to support normal VP growth. Transcriptomic analyses revealed that both EMS isolates shared overall expression patterns. Metabolic stress adaptation systems of non‐AHPND strain 2HP under SW and SSW were more upregulated than in AHPND strain 5HP. Specific virulence genes (i.e., *zot1* [zonula occludens toxin] and *vopS* [type III secretion effector]) and a general stress response gene (i.e., *rpoS* [stress response sigma factor]) were upregulated in strain 2HP under both SW and SSW. These expression profiles of strain 2HP suggest higher persistence, which might be useful for cell survival and non‐AHPND pathogenesis even without toxin production. We proposed that these genes encode virulence factor ‘candidates’ in non‐AHPND VP.

## Graphical abstract

To understand the disease‐causing characteristics of *Vibrio parahaemolyticus* in shrimp EMS, this comparative gene expression study analysed pathogenic (AHPND 5HP and non‐AHPND 2HP) and non‐pathogenic (S02) strains. Pathogenic strains shared similar expression profiles, distinct from the non‐pathogen. In nutrient‐depleted conditions, the PirAB^vp^ toxin‐lacking 2HP strain upregulated metabolic stress adaptation systems and specific virulence genes like *zot1*, *vopS* and *rpoS*. The findings suggest a link between metabolic adaptability and virulence, explaining pathogenesis without the primary toxin.

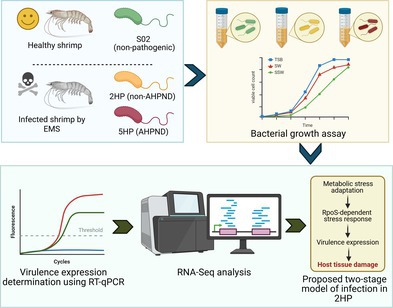

## 1 Introduction

Early mortality syndrome (EMS) is an emerging disease in the shrimp production industry. This disease is a serious problem worldwide for both *Penaeus monodon* and *Penaeus vannamei* farming (Restrepo et al. 2016; Soto‐Rodriguez et al. 2015; Zorriehzahra and Banaederakhshan 2015). *Vibrio parahaemolyticus* (VP) is the major cause of EMS (Soto‐Rodriguez et al. 2015; Tran et al. 2013). EMS‐causing VP is classified into two groups based on histological signs in infected shrimps: acute hepatopancreatic necrosis disease (AHPND) and non‐AHPND (Joshi et al. 2014). AHPND strains produce the toxin complex protein PirAB^vp^, causing necrotic tissues in the shrimp stomach and hepatopancreas by making pores in the host cell membrane (Han et al. 2015; Joshi et al. 2014; Lin et al. 2019). PirAB^vp^ toxin proteins are encoded by the *pirA* ^*vp*^ and *pirB* ^*vp*^ genes on a plasmid and act as virulence factors in AHPND isolates (Lee et al. 2015; Tran et al. 2013).

Shrimps infected by AHPND or non‐AHPND isolates commonly show the same gross signs, that is, empty stomach, pale hepatopancreas and empty midgut (Joshi et al. 2014; Prachumwat et al. 2019); however, the virulence factors in non‐AHPND are not identified. Non‐AHPND strains cannot produce the PirAB^vp^ toxins to perforate the host cells (Joshi et al. 2014). Yet, non‐AHPND infected shrimps could not eat and eventually die within a month (Prachumwat et al. 2019). Recently, we reported that, based on genomic analysis, a non‐AHPND VP strain 2HP carries a pre‐CTX prophage which contains *zot1* (zonula occludens toxin) and *ace1* (accessory cholera enterotoxin) genes (Thadtapong et al. 2020). This pre‐CTX prophage from 2HP is also found to be conserved in AHPND isolates (Thadtapong et al. 2020). Therefore, *zot1* and *ace1* might also be the virulence factor candidates in non‐AHPND and AHPND strains.

Changing environments can affect patterns of gene expression and lifestyles of bacteria (Feugeas et al. 2016). Specifically, environments where hosts are present could simulate virulence gene expression in pathogenic bacteria (Thomas and Wigneshweraraj 2014). A previous study has shown that expression levels of *pirA* ^*vp*^ and *pirB* ^*vp*^ in AHPND strains increased under the growth condition supplemented with shrimp tissue extracts. In this current study, we hypothesised that seawater and the presence of shrimps might induce some pathogenic characteristics in both AHPND and non‐AHPND VPs. In order to discover novel virulence factors specific to EMS strains (both AHPND and non‐AHPND VPs) and specific to non‐AHPND VP alone, a transcriptomic study was applied to identify the ‘overlapping’ expression in EMS VPs and ‘unique’ expression in AHPND and non‐AHPND VPs. The transcriptomic data from AHPND and non‐AHPND under seawater and spent seawater (i.e., filtered SW from shrimp cultivation) conditions could reveal the pattern of gene expression in response to the host living environment and the presence of the host in the environment, respectively, in pathogenic EMS VPs. The nutrient‐enriched condition represented by tryptic soy broth (TSB) plus 1.5% NaCl was also used in parallel to represent growth and expression profiles in the laboratory medium. We expect that the transcriptomic data from AHPND (strain 5HP) and non‐AHPND (strain 2HP) VPs in comparison to the non‐pathogenic VP (strain S02) under the studied conditions might reveal the factors or systems linked to EMS pathogenesis in addition to the known toxins. In‐depth information on gene expression in EMS isolates might be adapted to design diagnostic methods for EMS VP detection and prevention in shrimp production.

## 2 Materials and Methods

### 2.1 Bacterial Strains and Growth Condition

VP strains 5HP and 2HP were selected as models of AHPND and non‐AHPND strains, respectively (Joshi et al. 2014). VP S02 or the non‐pathogenic strain acted as a negative control. Three strains of VP were cultured in TSB supplemented with 1.5% NaCl and incubated at 30°C with shaking (200 rpm) overnight.

### 2.2 Water Samples

To prove that the presence of shrimps, potentially compounds secreted from shrimps, in seawater might induce expression of virulence factors in EMS‐causing VP strains, artificial seawater at a salinity of 25 ppt used to rear healthy shrimps for 4 days was sterilised through a 0.45 μm filter and was referred to as spent seawater herein. As a background control, artificial seawater was sterilised by filtration through a 0.45‐μm filter. Spent seawater and seawater were kept at 4°C until used. The spent seawater was sent to Environment & Laboratory company for checking the quality of water. Spent seawater sample was analysed following the physicochemical parameters in America Public Health Association (APHA 2012). The bacterial contamination was checked by cultivation of filtrates on TSA plates at 37°C, overnight.

### 2.3 Bacterial Growth Assay

Three strains of VP were cultured in TSB supplemented with 1.5% NaCl and incubated at 30°C with shaking overnight. Bacterial cultures were diluted 100‐fold in fresh TSB supplemented with 1.5% NaCl (5 mL). The diluted cultures were further incubated until OD_600_ of 0.6 and then distributed into two volumes: a 10‐fold dilution with TSB supplemented with 1.5% NaCl and non‐dilution. From the 10‐fold dilution, the cell suspensions were diluted further (100‐fold) into two conditions: Seawater and spent seawater. From non‐dilution, bacterial cell cultures were directly diluted into three conditions: TSB supplemented with 1.5% NaCl, seawater and spent seawater. All five conditions were incubated at 30°C with shaking at 200 rpm. Growth of each strain in each condition was monitored, and bacterial numbers were enumerated on TSA supplemented with 1.5% NaCl (Sieuwerts et al. 2008). The limitation of viable cell detection is 100 CFU/mL.

### 2.4 RNA Extraction

VP was cultured in three conditions: TSB supplemented with 1.5% NaCl, seawater and spent seawater. For RT‐qPCR experiments, bacterial cells were collected at six time points (0, 0.5, 1, 1.5, 2 and 4 h), representing lag, log and early stationary phases. For RNA‐seq analysis, bacterial cells were collected after 4 h of incubation. Transcriptional activity was stopped by adding 0.1 volume of 10% acid phenol pH 6.6 in absolute ethanol and stored at −80°C. RNA was extracted from 2 mL of samples. Large RNAs (> 200 nt) and small RNAs (< 200 nt) were separated by miRNeasy mini kit (Qiagen) and RNeasy MinElute kit (Qiagen). Genomic DNA (gDNA) contamination was removed from large and small RNA samples by DNase I treatment (Promega, USA). gDNA contamination was assessed by qPCR. Quantity and quality of large and small RNA were measured by Denovix DS‐11 FX+ spectrophotometer, Qubit RNA HS assay kit (Invitrogen, USA), and Bioanalyzer RNA nano 6000 chip (Agilent, USA).

### 2.5 Determination of Virulence Expression by Quantitative PCR (RT‐qPCR)

To investigate the virulence gene expression at each time point from different conditions, large RNA and small RNA were used and represent mRNA and small RNA levels, respectively. For toxin gene expression in 5HP, *pirA* ^*vp*^ and *pirB* ^*vp*^ genes encoding PirAB^vp^ toxin (Han et al. 2015) were selected as targets. For virulence gene expression in all three strains, *toxR* gene encoding a global transcription regulator of virulence (Ghenem et al. 2017) and *tlh* encoding a thermolabile hemolysin (Ghenem et al. 2017) were selected. For small RNA expression, Spot42 encoding a negative regulator of type III secretion system (Tanabe et al. 2015) and RyhB encoding a positive regulator of iron homeostasis and a negative regulator of motility (Tanabe et al. 2013) were selected. Ten nanograms of large RNA/small RNA were reverse transcribed into cDNA using miScript II RT kit (Qiagen). One hundred and fifty nanograms of cDNA was used to detect six known virulence factors of strain 5HP (*pirB* ^*vp*^, *toxR* and *tlh* for mRNA level, and Spot42 and RyhB for small RNA level) and four known virulence factors for strains 2HP and S02 (*toxR* and *tlh* for mRNA level, and Spot42 and RyhB for small RNA level) by QuantiNova Probe PCR kit (Qiagen). 16S and 5S rDNA genes were used as references for mRNA and small RNA levels, respectively. The primers and probes are listed in Table S1. To assess the level of gDNA contamination, the reaction mixture was prepared identically to that used for gene expression determination, with the exception that it did not contain reverse transcriptase enzyme. Any qPCR signal detected in this control reaction sample indicated the presence of contaminating gDNA, and the values were subtracted to reflect true mRNA level.

### 2.6 RNA‐Seq and Analysis

Total RNAs were extracted and sequenced through the service of Genomax (Singapore). Briefly, 100 ng of purified total RNA was used for library preparation. Ribosomal RNAs were removed with selective amplification by Ovation Complete Prokaryotic RNA‐Seq kit (NuGen, USA). RNA‐Seq libraries were sequenced on Illumina NovaSeq 6000 platform (Illumina, USA).

Raw data of RNA‐Seq were cleaned by Trim Galore and checked for the quality by FastQC (Andrews 2010; Krueger 2015). Trimmed reads from TSB condition were de novo assembled and analysed for differential expression (DE) by Rockhopper2 (Tjaden 2015) in order to compare among three strains, representing the expression values in RPKM (Reads Per Kilobase transcript per Million mapped reads). For comparisons within each EMS strain, draft genomes of 2HP and 5HP (Thadtapong et al. 2020; Yang et al. 2014) were used as reference genomes. To analyse RNA‐Seq data for the differential gene expression, trimmed reads were submitted on PATRIC server with Tuxedo strategy (Warren et al. 2015; Wattam et al. 2017). Briefly, trimmed reads were mapped to the draft genomes by Bowtie2 (Langmead and Salzberg 2012). Mapped reads were analysed for quality by SAMStat (Lassmann et al. 2011) and assembled by Cufflinks (Trapnell et al. 2010). Assembled transcripts were merged by Cuffmerge (Trapnell et al. 2010). DE analysis was performed using Cuffdiff, representing the expression values in FPKM (Fragments [similar to ‘Read’ in RPKM] Per Kilobase transcript per Million mapped reads) (Trapnell et al. 2010). The fold changes of expression were calculated in log‐ratio with two‐fold change set as a threshold. The *q*‐value less than 0.05 was considered as significant difference. The functional genes and systems were annotated by the RASTtk in PATRIC server (Brettin et al. 2015; Wattam et al. 2017). The virulence genes were additionally predicted by VFDB and Victors in PATRIC server (Liu et al. 2019; Sayers et al. 2019). The predicted prophage harbouring strain‐specific genes *zot1* and *ace1*, and *pirB* ^*vp*^ toxin (strain 5HP only) genes were included in the analysis. To explore the similarity of RNA‐Seq data, hierarchical clustering heatmap was applied to identify the pattern of dataset. In three strains comparison, trimmed reads were mapped, quality‐analysed, assembled as mentioned above. Merged assembled transcripts in GTF files from Cuffmerge were converted to counted number of reads by featureCount (Liao et al. 2013). The counted read data were used to analyse the similarity of dataset by hierarchical clustering heatmap via DESeq2 (Love et al. 2014). The featureCount and DESeq2 programs were run on the Galaxy RNA workbench 2.0 server (Fallmann et al. 2019). The transcriptomic data of three strains under TSB condition were analysed for the differential patterns of expression between pathogenic strains (2HP and 5HP) and non‐pathogenic strain (S02), and between AHPND (5HP) and non‐AHPND (2HP).

### 2.7 Statistical Analysis

Statistical analysis was calculated by SPSS Statistics 23. Virulence gene expressions in SW and SSW at each time point from qPCR data were analysed and compared by independent *t*‐test. Differential virulence expression of *pirB* ^*vp*^ from TSB condition was normalised to *T* = 0 min and analysed by paired *t*‐test. Virulence gene expression, except *pirB* ^*vp*^, in TSB at each time point from qPCR data were analysed by ANOVA followed by Tukey post hoc test. Time‐course gene expression levels were also analysed using ANOVA. *p*‐value less than 0.05 was considered a significant difference.

## 3 Results

### 3.1 Enriched Environment and Spent Seawater Condition Enhanced *V. parahaemolyticus* Growth

In order to explore the growth characteristics of VPs in different environments, each representative VP strain (i.e., non‐pathogenic S02, non‐AHPND 2HP and AHPND 5HP) was cultured in five different conditions (TSB plus 1.5% NaCl, seawater, spent seawater, seawater supplemented with 10% of TSB plus 1.5% NaCl, and spent seawater supplemented with 10% of TSB plus 1.5% NaCl). Growth characteristics were observed by enumeration using spot assay. The seawater and the spent seawater samples were sent to the Environment & Laboratory company (Thailand) for analysis. The results of water qualities were used as indicators for conditions of environment in the absence and presence of shrimps (Table S2).

**FIGURE 1:**
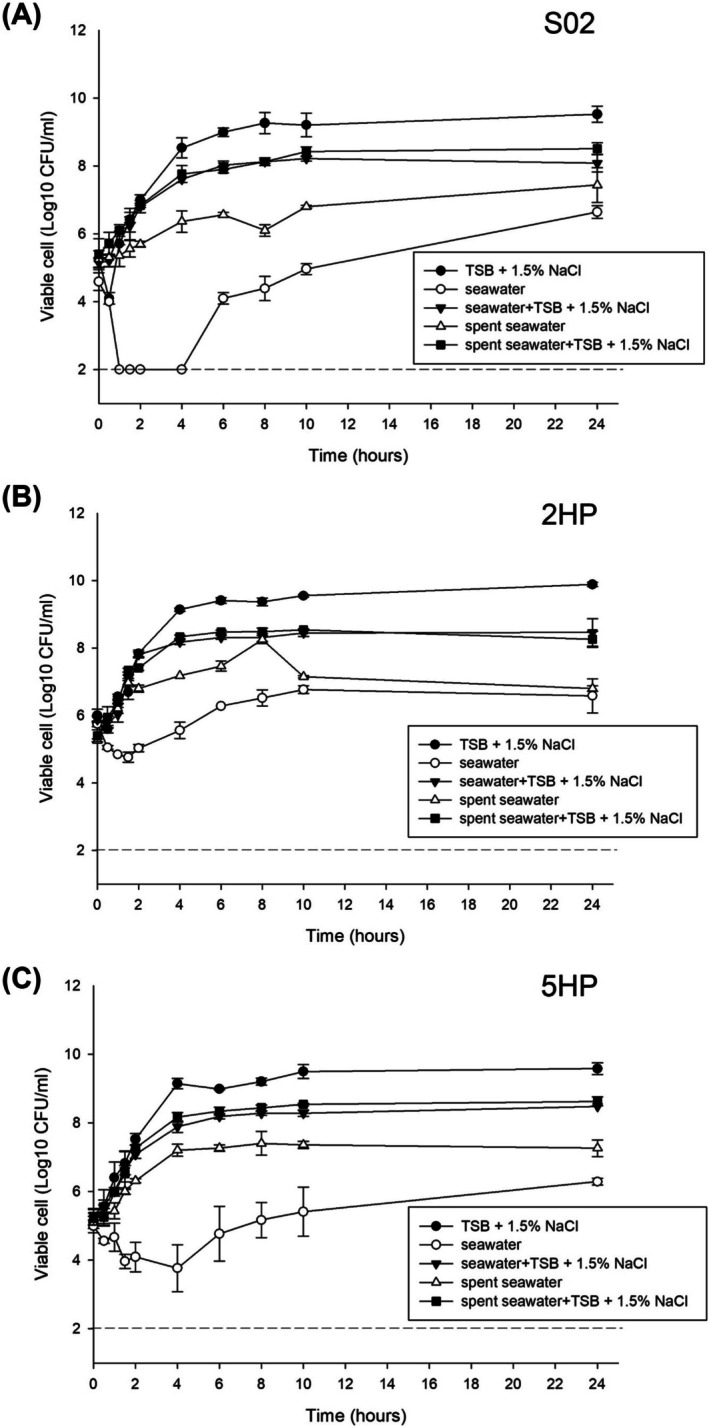
Growth characteristics of *V. parahaemolyticus* S02 (A), 2HP (B), and 5HP (C) under different conditions over 24 h. Closed circle: TSB plus 1.5% NaCl; closed inverted triangle: seawater with a trace of TSB plus 1.5% NaCl in culture inoculum; closed square: spent seawater with a trace of TSB plus 1.5% NaCl in culture inoculum; open circle: seawater; open triangle: spent seawater. Results are mean values and error bars represent standard deviation (replicates = 3). The limit of detection (100 CFU/mL) is indicated by the dashed line.

We found that all three strains of VP showed the same trend of growth in each condition (Figure 1, Table S3). TSB plus 1.5% NaCl was the best condition for growth in all three strains. Supplementation or trace of TSB plus 1.5% NaCl from the inoculum enhanced growth in seawater and spent seawater conditions. The results of water quality of spent seawater showed a high concentration of suspended solids and ammonia. Impaired growth was observed in conditions lacking TSB plus 1.5% NaCl. For example, S02 in seawater condition could not be detected at 0.5–6 h post‐inoculation. This could be due to the switch from actively growing VP to viable but non‐culturable (VBNC) form (Figure 1A). As a result, we continued the remaining experiments using the conditions that did not impair growth, that is, TSB plus 1.5% NaCl (labeled as “TSB”), seawater with TSB plus 1.5% NaCl (labeled as “SW”), and spent seawater with TSB plus 1.5% NaCl (labeled as “SSW”).

### 3.2 Virulence Gene Expression Increased in EMS‐Causing *V. parahaemolyticus* Under SSW

Growth characteristics of 2HP, 5HP and S02 strains in TSB, SW and SSW were compared to identify the time points representing different growth phases of the bacteria. We selected six time points that represented different growth phases: 0–0.5 h for lag phase, 0.5–2 h for log phase and 2–4 h for early stationary phase in SW, SSW and TSB (Figures S1 and S2). To study the effect of host (i.e., the presence of shrimps in shrimp‐conditioned SW) on virulence gene expression using transcriptomic analysis, we first identified the time points and conditions at which pathogenic bacteria (2HP and 5HP) expressed virulence factors. The RT‐qPCR technique was used to assess levels of gene expression at each time point/condition. However, virulence factors in non‐AHPND or other virulence factors in AHPND are unknown. Therefore, we used virulence factors of VP in the clinical field as indicators for the determination of virulence expression levels.

The 16S and 5S rDNA genes were initially selected to be reference genes for normalising the expression of coding RNA and non‐coding RNA levels, respectively. However, we found that levels of 16S and 5S rDNA transcripts significantly varied among strains and conditions over time points (Table S4). Levels of nutrients and minerals in SW and SSW might affect ribosomal synthesis in all strains. Therefore, for comparison of gene expression under SW and SSW conditions, 16S and 5S rDNA genes were considered unsuitable as reference genes for normalisation. Absolute numbers of transcripts were applied instead.

**FIGURE 2:**
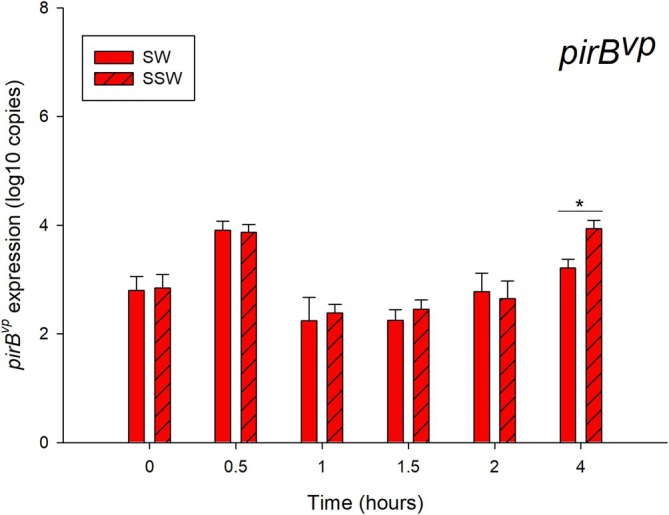
Expression levels of *pirB* ^*vp*^ from *V. parahaemolyticus* 5HP under SW and SSW conditions at each time point. Solid red and diagonally striped red bars represent SW and SSW conditions, respectively. * represents *p*‐value < 0.05 from independent *t*‐test. Results are mean values and error bars represent standard deviation in five independent experiments.

Only 5HP can produce PirAB^vp^ toxins, and the expression levels of *pirB* ^*vp*^, an expression reporter of the *pirAB* ^*vp*^ operon, between SW and SSW conditions were detected from this strain (Figure 2). 5HP showed equal levels of expression of *pirB* ^*vp*^ from SW and SSW at 0–2 h post‐inoculation. The *pirB* ^*vp*^ expression at 4 h post‐inoculation from SSW showed a significantly higher level than that in the SW condition.

**FIGURE 3:**
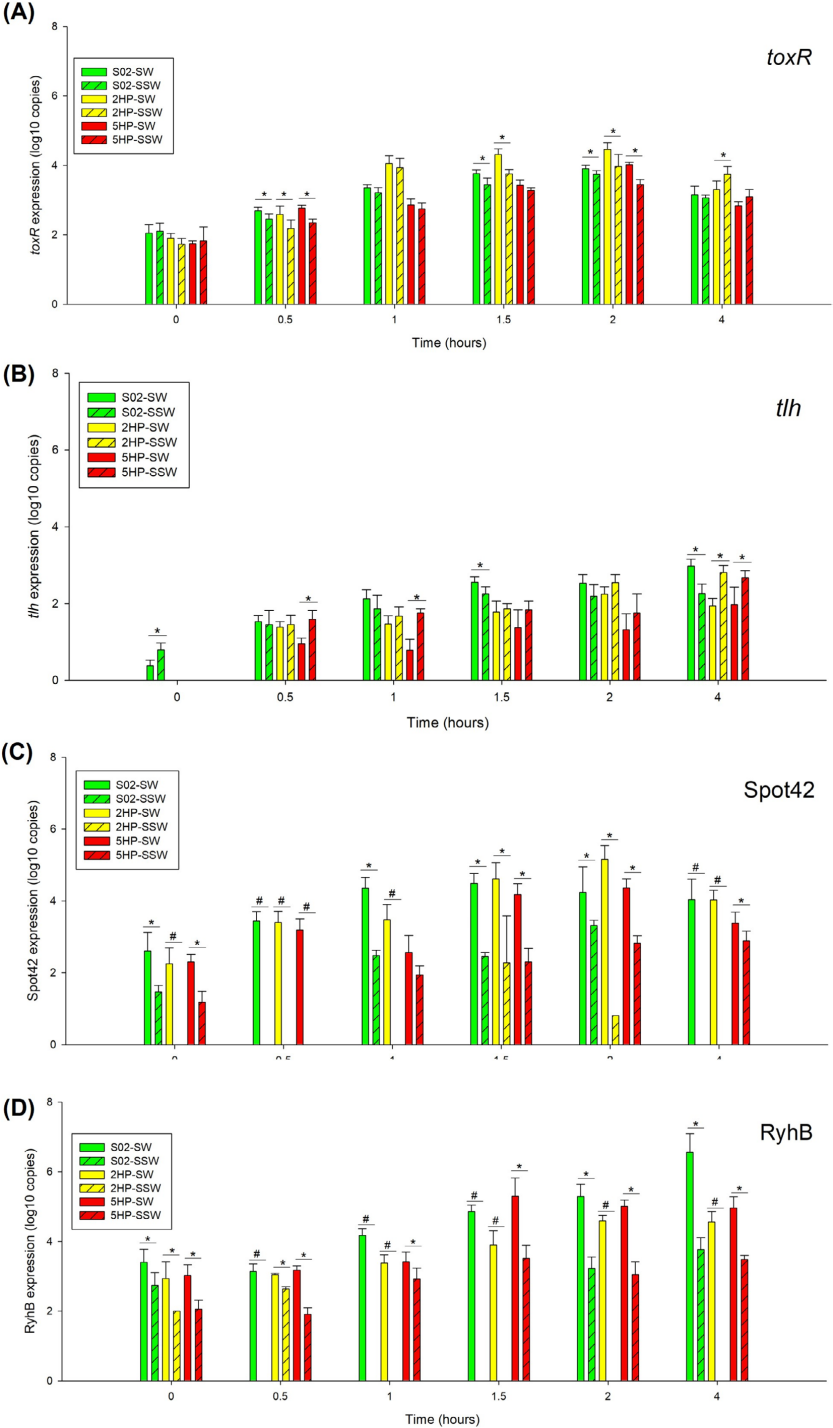
Expression levels of *toxR* (A), *tlh* (B), small RNA Spot42 (C), and RyhB (D) from *V. parahaemolyticus* S02, 2HP, and 5HP under SW and SSW conditions at each time point. Comparison of virulence expression from SW and SSW conditions in S02 (SW: solid green, SSW: diagonally striped green), 2HP (SW: solid yellow, SSW: diagonally striped yellow), and 5HP (SW: solid red, SSW: diagonally striped red) were presented in bar graphs. * represents *p*‐value < 0.05 from independent *t*‐test and # represents for only one condition is detectable. Results are mean values and error bars represent standard deviation in five independent experiments. No bars indicate non‐detectable signals by RT‐qPCR.

In order to monitor the expression levels among strains and conditions of selected virulence genes, we cultured S02, 2HP and 5HP in SW and SSW and analysed the gene expression by RT‐qPCR (Figure 3 and Table S4). Four genes were selected, that is, *toxR, tlh*, Spot42 and RyhB. As we hypothesised that SW and the presence of shrimps could induce pathogenic characteristics in EMS VP, we attempted to identify the time point at which the expression levels of selected genes were optimised for virulence characteristics. For the trends of *toxR* expression (Figure 3A), all three VP strains were expressing significantly higher levels in SW than in SSW. Only 2HP in SSW showed *toxR* expression at a significantly higher level than that in SW at 4 h post‐inoculation. In our results, the trend of *toxR* expression changed over the growth phases. For the trends of *tlh* expression (Figure 3B), expression levels in SSW were more than those in SW for 2HP and 5HP strains and peaked at 4 h post‐inoculation or early stationary phase. On the other hand, in S02 at *T* = 4 h, the expression of *tlh* under SW was higher than that under SSW.

Expression levels of non‐coding RNAs such as Spot42 and RyhB, which act as negative regulators for virulence characteristics, were expected to decrease in conditions and time points optimised for pathogenesis. Figure 3C shows, in all three strains, expression levels of Spot42 were detectable under SW conditions at every time point. Spot42 expression levels were lower in SSW conditions in all three strains at every growth phase. Spot42 levels in 2HP and 5HP strains under SSW were not detectable, that is, in 2HP at 0, 0.5, 1 and 4 h post‐inoculation and in 5HP at 0.5 h post‐inoculation. The pattern of small RNA RyhB expression was similar to that of Spot42 (Figure 3D). RyhB expression levels in 2HP, 5HP and S02 under SSW were significantly lower than those under the SW condition.

Generally, the 4‐h post‐inoculation time point consistently showed the expected pattern of virulence induction. At this time, the expression of key virulence genes like *pirB* ^*vp*^ and *tlh* peaked in the pathogenic strains (i.e., 5HP and 2HP) under SSW conditions (Figures 2C and 3B). Furthermore, the expression of other toxicity‐related genes was highest at 4 h, while negative regulators of virulence (i.e., Spot42 and RyhB) were at their lowest levels. This 4‐h time point was, therefore, selected for RNA collection and subsequent RNA‐Seq as it represented an early state of ‘persistence and readiness’ for infection. Additionally, this standardised *T* = 4 h condition allowed for a direct comparison of the transcriptomes between pathogenic and non‐pathogenic (i.e., S02) strains to better distinguish between virulence and a general stress response.

### 3.3 TSB Supports Better Virulence‐Associated Gene Expression Than SW and SSW

**FIGURE 4:**
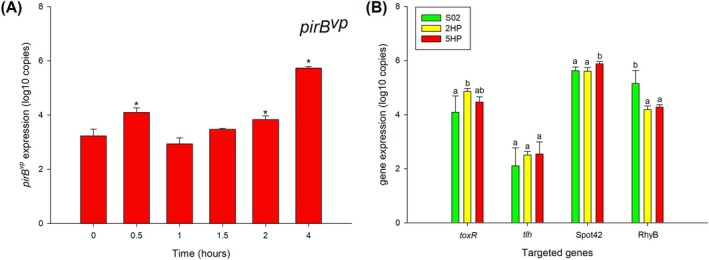
Expression levels of *pirB* ^*vp*^ (A) in *V. parahaemolyticus* 5H under TSB condition at each time point and *toxR*, *tlh*, small RNA Spot42 and small RNA RyhB from *V. parahaemolyticus* S02, 2HP and 5HP (B) under TSB condition at 4 h after inoculation. Green, yellow, and red represent *V. parahaemolyticus* S02, 2HP and 5HP, respectively. For *pirB* ^*vp*^, expression levels were compared to *T* = 0 min. * represents *p*‐value < 0.05 from independent *t*‐test. For *toxR*, *tlh*, small RNA Spot42 and small RNA RyhB, different letters over error bars indicate statistically significant difference within time point. Results are mean values and error bars represent standard deviation in five independent experiments.

To investigate expression levels of selected virulence genes in the enriched environment, we compared the expression profiles between pathogens (2HP and 5HP) and a non‐pathogen (S02), and between AHPND (5HP) and non‐AHPND (2HP) under growth in TSB. RT‐qPCR was used to assess the levels of *pirB* ^*vp*^ (strain 5HP only), *toxR, tlh*, Spot42 and RyhB. The *pirB* ^*vp*^ gene from 5HP was detected by RT‐qPCR (Figure 4A and Table S4). Expression levels of *pirB* ^*vp*^ increased over time. At 4 h post‐inoculation, *pirB* ^*vp*^ was expressed at its highest levels and was significantly different from *T* = 0. On the other hand, based on the results of immersion challenge (Joshi et al. 2014), we expected that expression levels of *toxR* and *tlh* in 5HP should be at their highest, followed by 2HP and S02, respectively. The *toxR* expression from all three strains is shown in Figure 4B. We found that expression levels of *toxR* in 2HP, 5HP and S02 increased over time (Figure S3). 2HP expressed *toxR* at a significantly lower level than 5HP and S02 at 0–0.5 h post‐inoculation. In 2HP, *toxR* expression increased and reached the highest level at 4 h post‐inoculation. In 5HP and S02, *toxR* expression increased until 2 h post‐inoculation but started to decrease at 4 h post‐inoculation. The *toxR* expression might be induced in lag and log phases (0–2 h post‐inoculation), then decreased expression might occur in the stationary phase. The *toxR* expression in 2HP still increased at 4 h post‐inoculation, while in 5HP and S02, *toxR* began to decrease (Figure 4B). This suggests a delayed response to high cell density and cell‐density‐related virulence in 2HP.

In addition, the expression levels of *tlh* were determined in all three strains (Figure 4B). We found that *tlh* expression in S02 was detected at *T* = 0. On the other hand, in 2HP and 5HP, *tlh* expression levels could be detected at 0.5‐h post‐inoculation (Figure S3). S02 expressed *tlh* at increasing levels until 1.5 h post‐inoculation; then *tlh* expression dropped. In 2HP and 5HP, *tlh* expression levels increased over time. S02 showed significantly higher *tlh* expression levels than those of 2HP and 5HP at 1–2 h post‐inoculation. The *tlh* expression levels from all three strains were not statistically different at 4 h post‐inoculation (Figure 4B). The results suggested that pathogen and non‐pathogen expressed *tlh* differently, at least in terms of different trends.

Similar to levels of expression under SW and SSW, we expected that the expression patterns of Spot42 and RhyB would decrease. 5HP should express the least amount, followed by 2HP and S02, respectively. The results showed that the trend of small RNA Spot42 expression in all three strains was the same, that is, increasing over the time points, similar to the *toxR* expression pattern (Figure S3). Unexpectedly, Spot42 from 5HP was expressed at the highest level at 4 h post‐inoculation. For small RNA RyhB, we found that RyhB in S02 and 2HP showed increasing expression levels over time (Figure S3). 5HP increased the expression of RyhB at 0–1.5 h post‐inoculation; then the expression dropped. At 4 h post‐inoculation, the RyhB level in S02 was higher than that in 2HP and in 5HP (Figure 4B). In summary, at 4 h post‐inoculation, the expression levels of virulence genes were upregulated in 2HP and 5HP when compared to those of S02, further supporting that 4 h post‐inoculation is a suitable time point to extract total RNAs for subsequent RNA‐seq experiment.

### 3.4 Transcriptomic Profiling of S02 Shows Different Patterns From Those of EMS‐Causing *V. parahaemolyticus*

**FIGURE 5:**
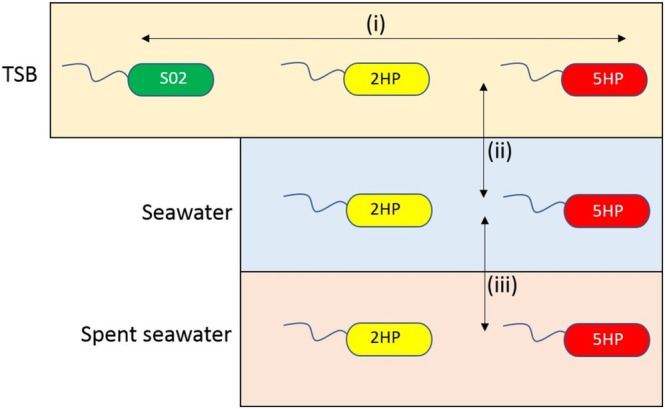
The flow chart of comparison in transcriptomic studies. Non‐pathogenic *V. parahaemolyticus* (S02) was compared to EMS‐causing *V. parahaemolyticus* (2HP and 5HP) (i). 2HP and 5HP were compare between TSB and seawater (SW) (ii) to study nutrient effect on differential gene expression. Another comparison, 2HP and 5HP were compared between seawater (SW) and spent seawater (SSW) (iii) to study the effect of presence of shrimp presence on differential gene expression. Green, yellow and red represent *V. parahaemolyticus* S02, 2HP and 5HP, respectively.

To investigate the effect of the presence of shrimp on gene expression in AHPND and non‐AHPND strains of *V. parahaemolyticus* and to identify novel virulence factors, we started by comparing the gene expression in the laboratory condition (TSB) to observe the natural characteristics between pathogen (2HP and 5HP) and non‐pathogen (S02) strains, and between AHPND and non‐AHPND. The flow chart for the transcriptomic study was shown in Figure 5. Transcriptomic analyses of 2HP and 5HP under the SW condition were compared with the TSB condition to explore DE between the laboratory condition and seawater in the ponds before culturing shrimps. Then, RNA‐Seq from 2HP and 5HP under the SSW condition was also compared with the SW condition to study the DE between the absence and presence of shrimps in the environment.

**FIGURE 6:**
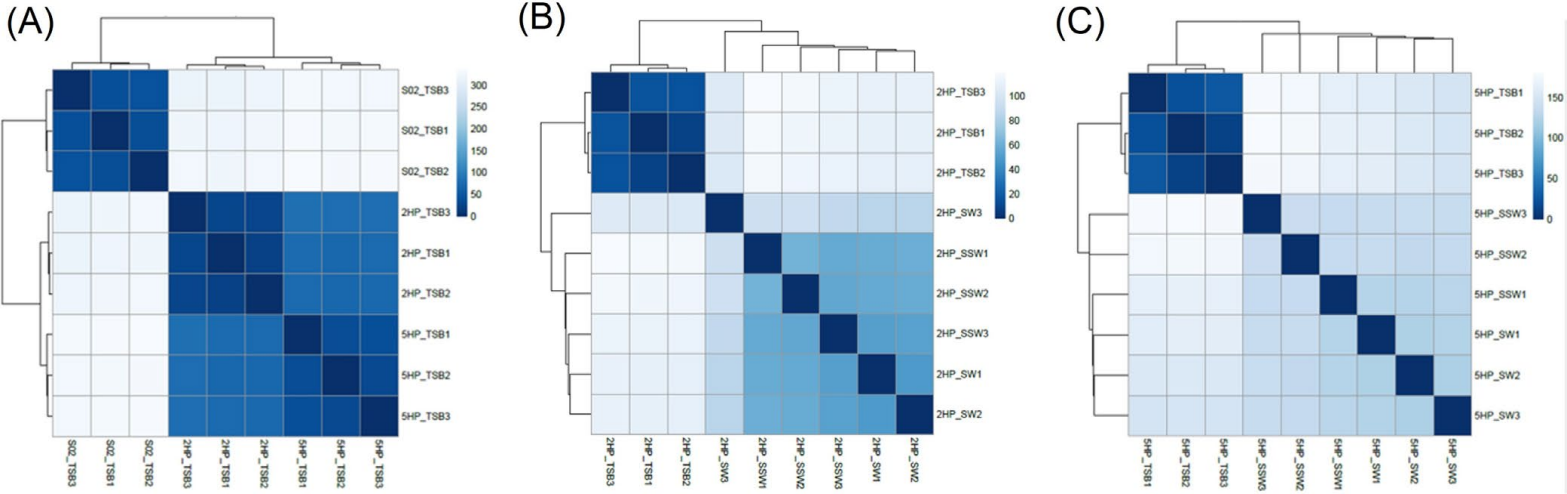
Sample‐sample distance heatmap from *V. parahaemolyticus* 2HP, 5HP and S02 under TSB condition (A), and *V. parahaemolyticus* 2HP (B) and 5HP (C) under TSB, SW and SSW conditions. Heatmaps were built on DESeq2 on normalised read counts with Euclidean distance between samples.

In order to characterise the natural characters of each isolate under enriched environment, transcriptomic analyses of 2HP, 5HP and S02 were performed. The heatmap showed that replicate samples within each strain were clustered, with S02 being separated from 2HP and 5HP (Figure 6A). Initial analysis suggested 2HP and 5HP shared some functions. EMS isolates might still share characteristics involved in pathogenesis under SW. A summary of transcriptomic data comparison from 2HP, 5HP and S02 is shown in Table 1. Total numbers of differentially expressed (DE) genes from 5HP versus S02 and 2HP versus S02 comparisons (or pathogenic vs. non‐pathogenic comparison) were 15.69% and 8.77% of total predicted genes from de novo assembly transcripts, respectively. On the other hand, total numbers of DE genes from the 5HP versus 2HP comparison were 5.48% of total predicted genes from de novo assembly transcripts. This data supported the results of the sample‐sample distance heatmap. 2HP and 5HP might share some functional genes.

**TABLE 1:** Summary of transcriptomic data of *V. parahaemolyticus* 2HP, 5HP and S02 under TSB condition.

	2HP versus S02	%^a^	5HP versus S02	%^a^	2HP versus 5HP	%^a^
Total DE genes	450	8.77	805	15.69	281	5.48
Total upregulated genes	354	6.90	503	9.81	147	2.87
Total downregulated genes	96	1.87	302	5.89	134	2.61
Total DE genes in functional categories	144	2.81	306	5.96	88	1.72
Upregulated genes	113	2.20	163	3.18	59	1.15
Downregulated genes	31	0.60	143	2.79	29	0.57
Total DE genes without functional categories	306	5.96	500	9.75	193	3.76
Upregulated genes	241	4.70	341	6.65	88	1.72
Downregulated genes	65	1.27	159	3.10	105	2.05

*Note:* Total predicted genes from de novo assembly transcripts are 5130 genes.

^a^ Percentage of differential gene expression in total predicted genes.

### 3.5 Transcriptomic Profiles of EMS‐Causing Under TSB Condition Are Different From SW and SSW Conditions

We found that data from SW and SSW were clustered. The sample‐sample distance heat map of 2HP and 5HP showed that data from the TSB condition were separate from the SW and SSW groups (Figure 6B,C). The SSW versus SW comparison from 2HP showed total DE genes at 2.45% of the total predicted genes (Table 2). On the other hand, total DE genes in the SW versus TSB and SSW versus TSB comparisons showed 25.74% and 28.04% of the total predicted genes (Table 2). This suggested that gene expression patterns within starved conditions (SW and SSW) were similar, while big differences were observed when compared to the enriched TSB. This pattern of transcriptomic data from 5HP under three different conditions showed similarity with 2HP. Total DE genes in the SW versus TSB and SSW versus TSB showed 24.31% and 24.59% of the total predicted genes. In contrast, the SSW versus SW comparison showed 2.54% of the total predicted genes. Again, this suggests that gene expression patterns in 5HP from the SW condition differed from SSW but still to a much lesser extent when compared to the TSB condition. The transcriptome profiles from 2HP and 5HP under TSB, SW, and SSW conditions support the sample‐sample distance heatmap.

**TABLE 2:** Summary of transcriptomic data of *V. parahaemolyticus* 2HP and 5HP under TSB, SW and SSW conditions.

	SW versus TSB	%^a^	SSW versus SW	%^a^	SSW versus TSB	%^a^
2HP						
Total DE genes	1390	25.63	133	2.45	1521	28.04
Total upregulated genes	857	15.80	4	0.07	730	13.46
Total downregulated genes	533	9.83	129	2.38	791	14.58
Total DE genes in functional categories	485	8.94	34	0.63	504	9.29
Upregulated genes	248	4.57	1	0.02	207	3.82
Downregulated genes	237	4.37	33	0.61	297	5.48
Total DE genes without functional categories	905	16.69	99	1.83	1017	18.75
Upregulated genes	609	11.23	3	0.06	522	9.62
Downregulated genes	296	5.46	96	1.77	495	9.13
5HP						
Total DE genes	1290	24.31	135	2.54	1305	24.59
Total upregulated genes	435	8.20	41	0.77	305	5.75
Total downregulated genes	855	16.11	94	1.77	1000	18.85
Total DE genes in functional categories	403	7.60	48	0.90	432	8.14
Upregulated genes	143	2.70	11	0.21	93	1.75
Downregulated genes	260	4.90	36	0.68	339	6.39
Total DE genes without functional categories	887	16.74	88	1.66	873	16.45
Upregulated genes	292	5.50	30	0.57	212	4.00
Downregulated genes	595	11.21	58	1.09	661	12.46

*Note:* Total predicted genes from de novo assembly transcripts for 2HP and 5HP are 5424 and 5306 genes, respectively.

^a^ Percentage of differential gene expression in total predicted genes.

### 3.6 Genes in Metabolism Category Are Most Affected by Strains and Conditions

The DE in specific functional categories was observed after comparison of transcriptomic data (Table 3). In functional categories, we found that genes in the metabolism category were among the most DE genes in every RNA‐Seq comparison (bolded number in Table 3). The metabolism category might play a role or assist the specific feature for pathogenic and non‐pathogenic phenotypes. Specifically, 2HP and 5HP in SW versus TSB and SSW versus TSB showed most of the significant DE genes in the metabolism category, followed by energy and protein processing, respectively. By comparing between host‐present (SSW) and host‐absent (SW) environments, we found that 2HP showed the highest number of DE genes in the categories of metabolism (15 genes), followed by membrane transport (5 genes). 5HP showed the highest DE genes in metabolism categories (18 genes) as well, followed by stress response, defence, and virulence (7 genes), and membrane transport (7 genes) categories.

**TABLE 3:** Summary of comparison of transcriptomic data from three different *V. parahaemolyticus* isolates and three different conditions.

Categories	Comparison		
	2HP versus S02	5HP versus S02	2HP versus 5HP
Cell envelope	4	3	0
Cellular processes	13	20	6
DNA processing	4	15	2
Energy	14	38	10
Membrane transport	17	30	4
Metabolism	**56**	**90**	**35**
Miscellaneous	1	3	3
Protein processing	6	64	20
Regulation and cell signalling	6	7	0
RNA processing	11	14	2
Stress response, defence and virulence	12	22	6
No functional categories	306	499	193
Total	450	805	281

Categories	Comparison		
	2HP, SW versus TSB	2HP, SSW versus TSB	2HP, SSW versus SW
Cell envelope	12	11	1
Cellular processes	28	27	3
DNA processing	15	20	2
Energy	90	84	3
Membrane transport	57	57	5
Metabolism	**157**	**186**	**15**
Miscellaneous	4	4	1
Protein processing	72	61	1
Regulation and cell signalling	5	1	0
RNA processing	9	14	0
Stress response, defence and virulence	36	39	3
No functional categories	905	1017	99
Total	1390	1521	133

Categories	Comparison		
	5HP, SW versus TSB	5HP, SSW versus TSB	5HP, SSW versus SW
Cell envelope	10	13	4
Cellular processes	30	37	1
DNA processing	14	26	1
Energy	70	68	4
Membrane transport	33	38	7
Metabolism	**153**	**141**	**18**
Miscellaneous	3	4	1
Protein processing	42	54	3
Regulation and cell signalling	2	2	0
RNA processing	9	11	1
Stress response, defence and virulence	37	38	7
No functional categories	887	873	88
Total	1290	1305	135

*Note:* The bolded numbers indicate the most DE genes in corresponding RNA‐Seq comparison.

### 3.7 Shared and Unique Features Among AHPND and Non‐AHPND Strains

**FIGURE 7:**
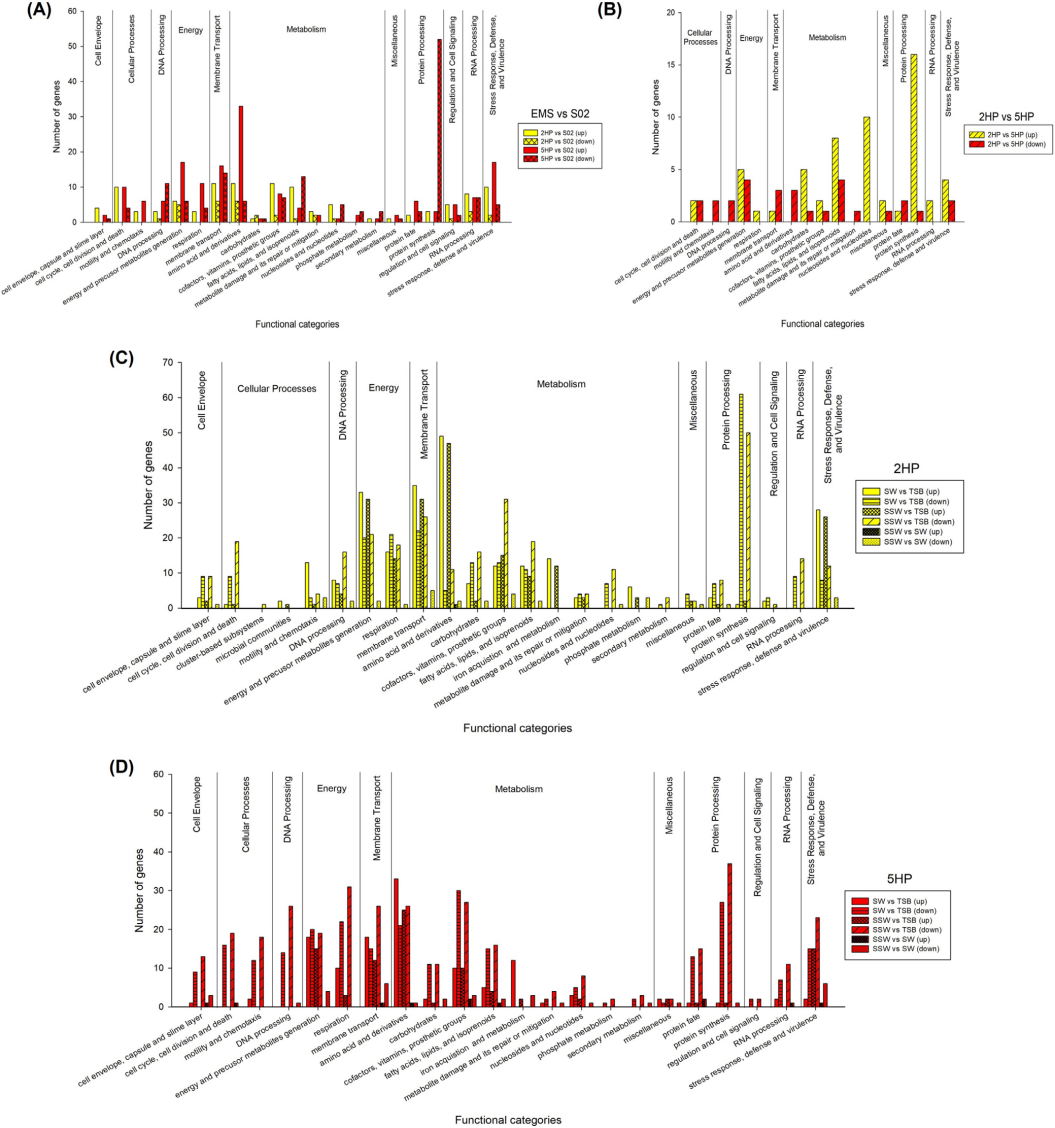
Distribution of genes of differential expression in functional categories, predicted by RASTtk. Comparisons of EMS versus S02 (A), 2HP versus 5HP (B), three different conditions in 2HP (C) and 5HP (D) showed numbers of upregulated and downregulated genes in each functional category. (A) The numbers of upregulated and downregulated genes of 2HP versus S02, and the numbers of upregulated and downregulated genes of 5HP versus S02 represented in solid yellow, crossed yellow, solid red, and crossed red bars, respectively. (B) The numbers of upregulated and downregulated genes of 2HP versus 5HP represented in diagonally striped yellow and diagonally striped red bars, respectively. The numbers of DE genes of 2HP and 5HP from three comparisons are shown in yellow and red in panel C and D, respectively; upregulated (solid bar) and downregulated (horizontally striped bars) genes from SW versus TSB, upregulated (crossed striped bars) and downregulated (diagonally striped bars) genes from SSW versus TSB, and upregulated (black dotted background bars) and downregulated (dotted bars) genes from SSW versus SW.

**FIGURE 8:**
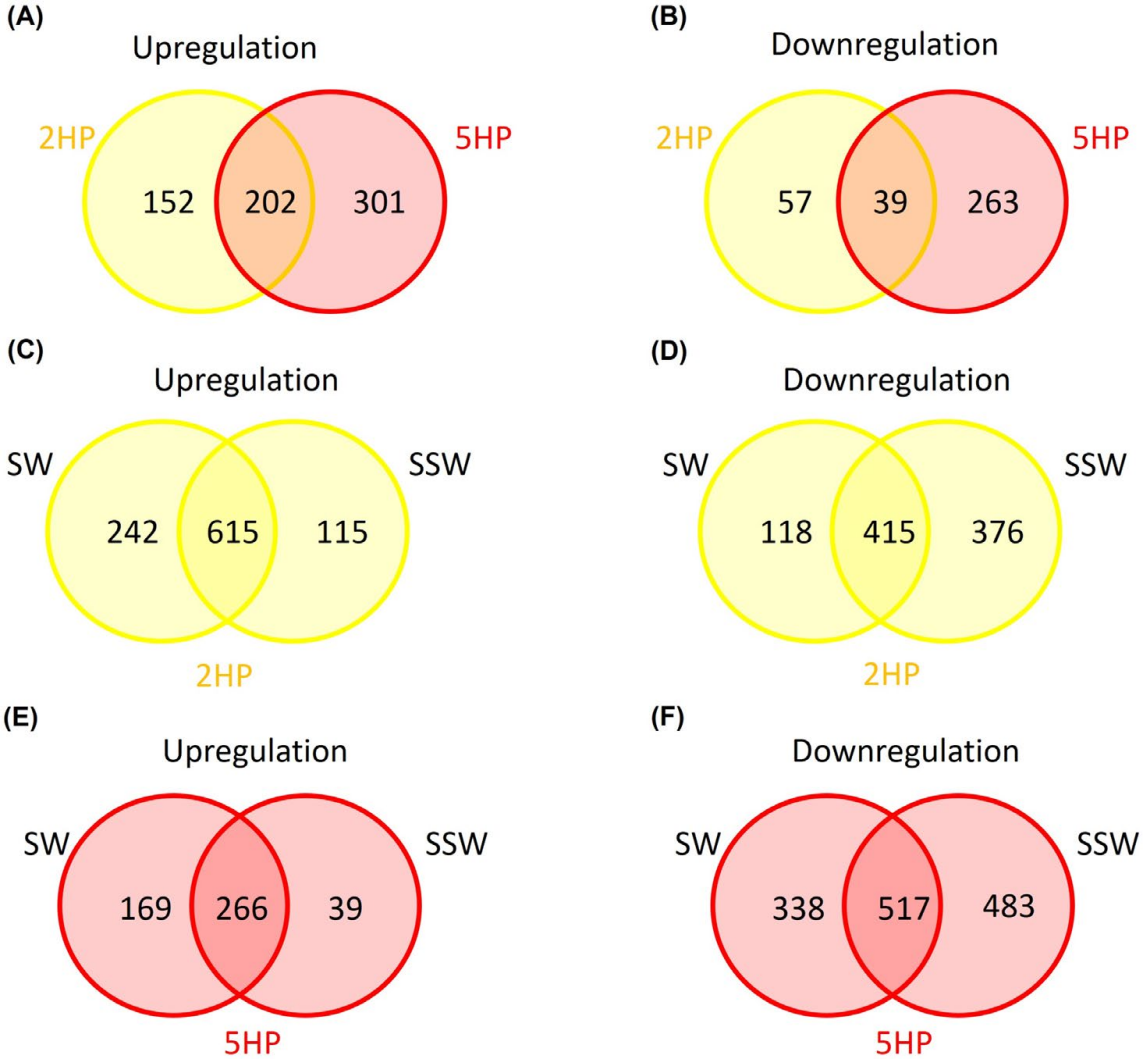
Two‐circle Venn diagrams of distribution showing unique and overlapped DE genes from EMS‐causing strains compared with S02 (A, B) in TSB. EMS‐causing *V. parahaemolyticus* 2HP (C, D) and 5HP (E, F) under SW and SSW compared with TSB. Yellow and red colours represent 2HP and 5HP, respectively. The overlapped areas represent the shared upregulated/downregulated genes with numbers of genes among strains or conditions.

In order to categorise the unique and common features of DE genes among EMS strains, the upregulated and downregulated genes in each category were explored and shown in Figure 7 and Tables S5–S13. Shared and unique upregulated/downregulated genes in EMS isolates are shown in Figure 8. With EMS versus S02, we found that EMS isolates shared upregulated systems in many functional categories (Figure 7A). Seven categories in five different super‐classes are shared between 2HP and 5HP (yellow and red bars in Figure 7A and Table S14). Changes in expression levels of metabolism genes were strain‐specific. 5HP showed downregulation in 52 genes in the protein synthesis system in protein processing superclass (Figure 7A). A comparison between 2HP and 5HP revealed that a number of metabolism genes and protein synthesis genes in 2HP were higher than in 5HP (diagonally striped yellow bars in Figure 7B).

Comparisons between EMS strains in depleted (SW and SSW) versus repleted (TSB) conditions are shown in Figure 7C,D, respectively. The total numbers of upregulated (972 genes) and downregulated (909 genes) genes in SW and SSW in 2HP are close (Figure 8C,D and Table S15). The overlapped upregulated genes are 615 genes from nine different functional categories while the overlapped downregulated genes are 415 genes from 11 different functional categories. The categories with upregulated expression in SW include flagellum (cellular processes), choline uptake and conversion to betaine cluster (stress response, defence and virulence), and conjugative transfer. In SSW, we found that ferrous iron transport genes were upregulated. *toxR* expression did not show significant change but *tlh* was upregulated in SW versus TSB and SSW versus TSB, not SSW versus SW (Tables S16 and S17). The increasing Spot42 expression from RT‐qPCR (Figure 3C) did not correlate with T3SS1 (upregulated *vopR, vscD* and *exsA* in SSW vs. SW) (Table S17). Spot42 in 2HP positively affected T3SS1, as also observed in 5HP. Vibrioferrin synthesis and iron uptake system showed shared upregulation in SW and SSW (Table S15); they did not correlate with the expression of RyhB from RT‐qPCR results (Figure 3D). Unlike in 5HP, RyhB in 2HP may not control vibrioferrin production. Overall, from RNA‐Seq in 2HP, we found that 2HP upregulates some categories, that is, energy and precursor metabolism generation (energy), membrane transport, amino acid and derivatives (metabolism), iron acquisition and metabolism (metabolism), phosphate metabolism (metabolism), and stress response, defence and virulence (Figure 7C). 2HP could require these genes to survive and respond to nutrient‐limiting conditions. As genes in stress response and iron uptake could be considered as virulence factors for pathogenic *V. parahaemolyticus* strains (Ghenem et al. 2017), 2HP could possibly use both categories to establish EMS pathogenesis, instead of PirAB^vp^ toxin.

Limited nutrient also showed extreme effects on cellular and molecular mechanisms of 5HP (Figure 7D) under SW and SSW conditions. There are 266 overlapped upregulated genes in SW and SSW conditions from 6 different functional categories and 517 overlapped downregulated genes from 11 different functional categories (Figure 8E,F and Table S18). SW condition upregulated vibrioferrin synthesis (iron acquisition, metabolism), heme O and heme A biosynthesis (energy), and oxidative and osmotic stress response (stress response, defence and virulence), while SSW condition upregulated genes encoding hydroxymethylpyrimidine (HMP) ABC transporter while downregulated KDO2‐lipid A biosynthesis and outer membrane (cell envelope), cell division (cellular processes), motility and chemotaxis (cellular processes), DNA repairing (DNA processing), type IV and type VI secretion systems (membrane transport), protein fate and protein synthesis (protein processing), RNA processing, and stress response, defence and virulence. *toxR* results from RT‐qPCR (Figure 3A) agreed with those of RNA‐Seq (i.e., no significant change); however, *tlh* results did not (Figure 3B). The expression of Spot42 in RT‐qPCR was increased in SW (Figure 3C), which did not agree with T3SS1 upregulation. RNA‐Seq comparison between SSW and SW revealed that *vscR*, *vscQ* and *vscP* genes (encoding T3SS1; Hotinger et al. 2021; O'Boyle and Boyd 2014) were upregulated in SW (Table S17). Spot42, in this case, could play a positive effect for T3SS1 expression. RyhB expression was increased in SW in RT‐qPCR results (Figure 3D); this correlated with upregulation of vibrioferrin synthesis in RNA‐Seq (Table S18). From RNA‐seq data, 5HP might adapt by slowing down the metabolic rate and persisting in SW and SSW.

We also analysed DE virulence genes for determining the candidate virulence factor(s) in 2HP and new virulence factor(s) in 5HP besides *pirAB* ^*vp*^. In 2HP, six virulence‐associated genes were upregulated in both SW and SSW (*sucA*, *zot1*, *vopS*, *tlh*, *VC2647* and *rpoS*) (Table S16). These genes encoded 2‐oxoglutarate dehydrogenase E1 component in TCA cycle (*sucA*; Miyakoshi et al. 2018), zona occluden toxin 1 from pre‐CTX prophage (*zot1*; Thadtapong et al. 2020), T3SS1 effector (*vopS*; R. Wang, Zhong, et al. 2015), thermolabile hemolysin toxin (*tlh*; Ghenem et al. 2017), transcriptional regulator in PadR family (*VC2647*; Q. Wang, Millet, et al. 2015), and alternative sigma factor for stress response (*rpoS*; Dong and Schellhorn 2010). Four of the six upregulated virulence‐associated genes directly link to pathogenic characteristics (*zot1*, *vopS, tlh* and *rpoS*), especially *zot1*, which is a 2HP‐specific gene. These genes could be virulence factors in 2HP that respond to starvation conditions. No virulence gene was induced in SSW‐specific conditions (Table S15). Host‐presence condition (SSW) did not affect virulence expression in 2HP. In 5HP, four virulence genes showed upregulation in SW and SSW (*bioB*, *guaB*, *tlh* and *VC2647*). *bioB* and *guaB* genes encode biotin synthase in biotin synthesis (Choi‐Rhee and Cronan 2005) and inosine‐5′‐monophosphate dehydrogenase in xantosine 5′‐phosphate biosynthesis (Zhou et al. 1997), respectively. Two virulence genes are upregulated in SW and shared in 2HP and 5HP (*tlh* and *VC2647*).

## 4 Discussion

### 4.1 Water Quality Analysis

Previous study showed that at day 4 post‐infection, shrimps infected with 5HP and 2HP VPs showed 100% and 75% mortality rates, respectively (Joshi et al. 2014). To investigate the qualities of fresh seawater and the shrimp‐conditioned seawater in light of identification of potential compounds secreted from shrimps and the effect of shrimps on expression of EMS virulence factors in EMS‐causing VPs, SW and SSW (on day 4) were used. The SSW contained considerably higher concentration of suspended solids and ammonia in comparison to the fresh SW. The water quality of SSW was above the target water standard for shrimp farm wastewater recommended by the Global Aquaculture Alliance (≤ 50 mg/L for total suspended solid and ≤ 3 mg/L for total ammonia; Boyd and Gautier 2000). The increased suspended solids were most likely waste from the shrimps, for example, unconsumed feed, faeces and urine (Boopathy and Lyles 2008; Chen et al. 2015). High suspended solids and ammonia concentration in SSW can serve as potential nutrients and a nitrogen source (Urdaci et al. 1988), respectively, and benefit growth in all three VP strains. The growth characteristics for all three strains in SSW were to some extent comparable to their growth in the rich medium TSB. This suggests that waste products from shrimp cultivation are sufficient to support normal growth of VP.

### 4.2 Differential Gene Expression in 2HP and 5HP

**FIGURE 9:**
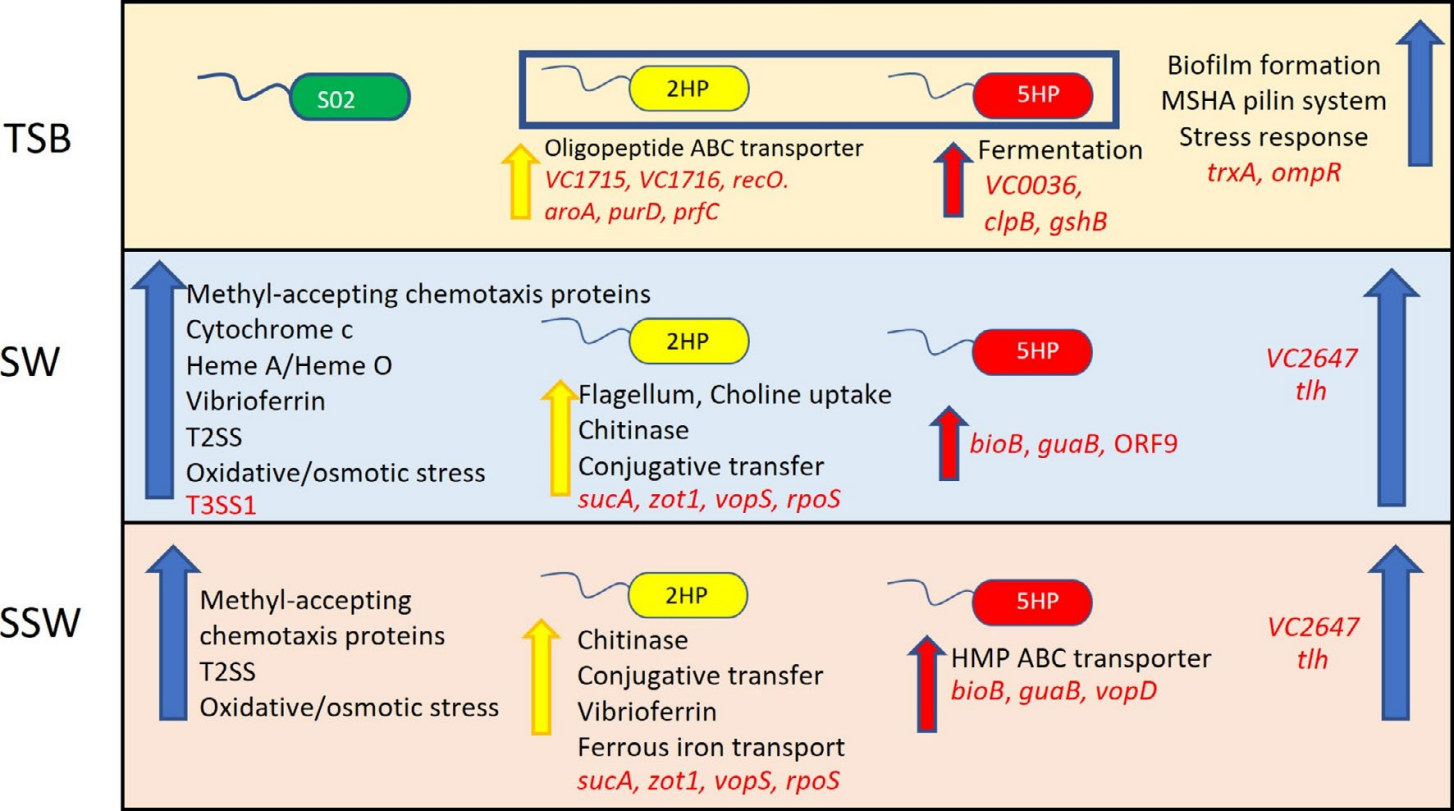
Summary of transcriptomic data. S02, 2HP and 5HP represented in green, yellow, red cells, respectively. The overlapped upregulated genes/systems in 2HP and 5HP represent in blue arrows. The unique upregulated genes/systems in 2HP and 5HP are shown in yellow and red arrows, respectively. Name of upregulated systems and virulence‐associated genes are shown in black and red, respectively.

To study DE genes in 2HP and in 5HP between enriched and starved conditions, transcriptomic data from TSB, SW and SSW conditions were compared. Figure 9 summarises the transcriptomic data from this study. In 2HP, the oligopeptide ABC transporter, which plays a role in adhesion, biofilm formation and hemolytic activity, was upregulated (Liu et al. 2017). In our study, upregulated virulence genes in 2HP and 5HP were of different functional categories. When 2HP and 5HP were exposed to SW and SSW, methyl‐accepting chemotaxis proteins (MCPs), type II secretion system (T2SS) and oxidative stress response were upregulated. Moreover, *VC2647* and *tlh* expression levels were increased in SW and SSW; both genes could be virulence factor candidates in EMS. T2SS is a channel for secretion of toxin in pathogenic bacteria (Korotkov et al. 2012). EMS strains could use T2SS for toxin secretion to hosts or environments. Expression of iron acquisition (cytochrome c, heme A/heme O and vibrioferrin) and T3SS1 were increased in EMS in only SW. Chitinase, conjugative transfer (*trbB*, *trbG*, *trbF* and *trbL*) or T4SS, and four virulence‐associated genes (*sucA*, *zot1*, *vopS* and *rpoS*) were upregulated in 2HP in SW and SSW. T4SS is a channel for horizontal gene transfer in AHPND (Wang et al. 2022), suggesting non‐AHPND like 2HP might transfer genetic materials in the same pathway as AHPND. Toxin gene *zot1*, chitinase gene, T3SS1 effector (*vopS*), and alternative sigma factor for general stress response (*rpoS*) are directly linked to pathogenic characteristics and could be virulence factor candidates in 2HP, instead of the PirAB^vp^ toxin. The induction of both the general stress response (regulated by RpoS) and specific virulence factors (e.g., Zot1 toxin and VopS effector) suggests a two‐stage model of infection where an initial RpoS‐dependent stress response confers the metabolic fitness required for the subsequent deployment of specific virulence factors that cause tissue damage and mortality (Table 4).

**TABLE 4:** The proposed two‐stage model of infection in *V. parahaemolyticus* 2HP.

Stage	Process	Mechanism	Outcome
Stage 1	Activation of metabolic fitness and adaptation	RpoS‐dependent stress response	Increased intra‐host survival and infectivity
Stage 2	Virulence factor deployment	Expression of specific virulence factors	Established infection; host tissue damage

As the virulence factor(s) of 2HP is still unknown, DE genes among VPs that could infer virulence or persistence, and therefore increased chance to initiate disease, were determined (Table S19). We found that *trxA* and *ompR* were upregulated in both 2HP and 5HP. *trxA* encodes thioredoxin in oxidative stress response (May et al. 2019) and *ompR* encodes the two‐component systems (TCS) regulator OmpR in osmotic stress response (Flores‐Kim and Darwin 2016). EMS could use both functions to survive under oxidative and osmotic stresses. Upregulated genes involved in 2HP virulence consisted of cell division genes *VC1715* and *VC1716* (Merrell et al. 2002), DNA processing gene *recO* (Kidane et al. 2004), metabolism in amino acids and derivative gene *aroA* (Duncan et al. 1984), metabolism in nucleosides and nucleotides gene *purD* (Aiba and Mizobuchi 1989), and protein processing gene *prfC* (Mikuni et al. 1994). In 5HP, we identified three virulence genes that were upregulated, that is, *VC0036*, *clpB* and *gshB*. These genes are involved in acid (*clpB*, *gshB*) and oxidative (*VC0036*, *clpB*) stresses (Alam et al. 2021; Conner et al. 2016; Nag et al. 2005; Wang et al. 2018).

### 4.3 Major Differential Response Is in Metabolism Processes

As we identified that metabolism is the largest category that responded to changing environment and potentially starved condition in the presence of host (i.e., SSW), our finding suggested the EMS‐causing VPs reprogram for nutrient uptake and enhance their ability to cause the disease. In RNA‐Seq results, the pattern of metabolism expression in 2HP and 5HP was similar. Nutrient uptake systems in 2HP and 5HP might be linked to virulence expression for EMS pathogenesis.

The dTDP‐glucose 4,6‐dehydrogenase and glucose‐1‐phosphatethymidyl transferase genes in the cell envelope showed upregulated expression in 2HP and 5HP. Both genes play a role in capsule and S‐layer systems, involving biofilm formation (Flemming 2016; Gerbino et al. 2015). We also found that expression levels of the mannose‐sensitive haemagglutinin (MSHA) pilin system, MSHA biogenesis, and negative regulator of flagellin synthesis FlgM were increased in EMS isolates (Table S12). MSHA or type IV pilus involves surface anchoring (Floyd et al. 2020), suggesting better swarming, surface attachment and biofilm formation in EMS VPs than in S02. Expression levels of cell division genes were increased in EMS VPs, suggesting an effect on cell replication. This data correlated with the growth characterisation of EMS strains as they did not show VBNC state in SW (no supplement with TSB, Figure 1). Both adhesion/biofilm formation and colonisation exhibit an association with EMS pathogenesis initiation. On the other hand, in the stress response system, we found that expression levels of aerotolerance protein BatA/BatC and BatD, and Envz/OmpR TCS were upregulated in EMS VPs. Aerotolerance proteins respond to reactive oxygen species (ROS) or oxidative stress (Stewart et al. 2012), and Envz/OmpR TCS responds to osmotic stress (Tipton and Rather 2017). EMS VPs might apply these systems to accordingly respond to oxidative and osmotic stressors. Interestingly, the most downregulated expression in the overlapped area contains Delta 1‐pyrroline‐5‐carboxylate dehydrogenase domain protein genes in the metabolism (amino acid derivatives) system; the EMS VPs might switch to decelerate the translation step and ribosomal hibernation in a starved environment, a strategy pathogens use to enhance survival and pertain pathogenesis (Prossliner et al. 2021; Wang et al. 2020).

## 5 Conclusion

By comparing the transcriptomic data of each strain and condition, we aimed to identify differences in the molecular systems that control the phenotypes of pathogenic (AHPND and non‐AHPND) and non‐pathogenic VPs. We also used this data to predict virulence factor candidates in the 2HP strain, for which the pathogenic mechanism was previously unknown. Prior to RNA‐Seq analysis, we accessed the growth characteristics of 2HP, 5HP and S02 in five different conditions. We found that TSB plus 1.5% NaCl was the most suitable condition for growth, and supplementing SW and SSW with TSB plus 1.5% NaCl improved bacterial growth. Subsequently, expression patterns of virulence‐associated genes were measured by RT‐qPCR. We observed that 2HP and 5HP strains showed peak expression at the 4‐h post‐inoculation, and this time point was therefore selected for the subsequent RNA‐Seq analysis. From RNA‐Seq results, we compared the three strains in TSB to identify DE genes between EMS strains and the S02 strain. We also compared the three conditions (TSB, SW and SSW) within EMS strains to find DE genes under nutrient‐rich and starved conditions. Across all comparisons, genes related to metabolism showed the greatest changes, suggesting a link between nutrient uptake and virulence (Abu Kwaik and Bumann 2013). Our findings indicated that the pathogenic 2HP and 5HP shared similar expression patterns, particularly for genes involved in stress response and cellular processing. Under starved conditions, 2HP and 5HP showed increased expression of genes involved in iron acquisition, chemotaxis, secretion systems, and stress response. The expression patterns in SW and SSW were largely similar, suggesting that simulated host presence did not significantly alter virulence expression. Notably, the genes *VC2647* and *tlh* were upregulated in SW and SSW in both pathogenic strains, identifying them as potential new virulence factors. Specifically, for the 2HP strain, we observed upregulation of iron uptake systems and several virulence‐associated genes, including *sucA*, *zot1*, *vopS* and *rpoS*. We propose that these systems and genes could be essential for mediating the pathogenesis in 2HP, even in the absence of the PirAB^vp^ toxin.

## Author Contributions

**Nalumon Thadtapong:** conceptualization, methodology, formal analysis, validation, visualization, writing – review and editing, writing – original draft. **Varodom Charoensawan:** conceptualization, methodology, investigation, writing – review and editing. **Vanvimon Saksmerprome:** conceptualization, methodology, resources, writing – review and editing. **Soraya Chaturongakul:** conceptualization, methodology, supervision, funding acquisition, writing – review and editing.

## Conflicts of Interest

The authors declare no conflicts of interest.

## Supporting information


**Figure S1:** Comparison of growth characteristics of three strains of *V. parahaemolyticus* in SW and SSW.
**Figure S2:** Comparison of growth characteristics of three strains of *V. parahaemolyticus* in TSB plus 1.5% NaCl.
**Figure S3:** Expression levels of *toxR* (A), *tlh* (B), small RNA Spot42 (C) and RyhB (D) from *V. parahaemolyticus* S02, 2HP and 5HP under TSB condition at each time point.

**Table S1:** List of primers and probes.
**Table S2:** Water qualities of seawater and spent seawater.
**Table S3:** Time coursing of *V. parahaemolyticus* growth under five different conditions.
**Table S4:** Expression levels of selected virulence factors from *V. parahaemolyticus* 2HP, 5HP and S02 under three different conditions.
**Table S5:** List of differential expression genes when compared 2HP versus S02.
**Table S6:** List of differential expression genes when compared 5HP versus S02 2HP versus S02.
**Table S7:** List of differential expression genes when compared 2HP versus 5HP.
**Table S8:** List of differential expression genes in 5HP when compared SW versus TSB.
**Table S9:** List of differential expression genes in 5HP when compared SSW versus TSB.
**Table S10:** List of differential expression genes in 5HP when compared SSW versus SW.
**Table S11:** List of differential expression genes in 2HP when compared SW versus TSB.
**Table S12:** List of differential expression genes in 2HP when compared SSW versus TSB.
**Table S13:** List of differential expression genes in 2HP when compared SSW versus SW.
**Table S14:** List of overlapped and unique upregulated/downregulated genes when comparing each EMS strain to non‐pathogenic S02.
**Table S15:** List of overlapped and unique upregulated/downregulated genes when comparing transcriptomic data of 2HP under SW, SSW and TSB conditions.
**Table S16:** List of overlapped and unique upregulated/downregulated virulence genes when comparing transcriptomic data of 2HP and 5HP under SW, SSW and TSB conditions.
**Table S17:** List of differentially expressed virulence genes in 2HP and 5HP when compared between SSW versus SW.
**Table S18:** List of overlapped and unique upregulated/downregulated genes when comparing transcriptomic data of 5HP under SW, SSW and TSB conditions.
**Table S19:** List of overlapped and unique upregulated/downregulated virulence genes when comparing transcriptomic data among 2HP, 5HP and S02 in TSB conditions.
**Table S20:** List of SRA accession number.

## Data Availability

The RNA‐Seq data for three strains of *V. parahaemolyticus* under TSB plus 1.5% NaCl condition have been deposited to GenBank BioProject accession numbers PRJNA628080, PRJNA628841, and PRJNA627978, for 2HP, 5HP, and S02, respectively. The RNA‐Seq for 2HP under SW and SSW have been deposited to GenBank BioProject accession number PRJNA628148 and PRJNA628564, respectively. The RNA‐Seq data for 5HP under SW and SSW have been deposited to GenBank BioProject accession number PRJNA629291 and PRJNA629606, respectively. List of SRA accession number showed in Supplementary Table S20.
