# Supplementary material for: Pathogenic Characteristics of Shrimp Early Mortality Syndrome (EMS)‐Causing Vibrio parahaemolyticus: A Comparative Transcriptomic Study Suggests the Relationship Between Metabolic Fitness and Virulence Gene Expression
Source: Environ Microbiol Rep. 2025 Nov 25;17(6):e70219. doi: 10.1111/1758-2229.70219 (PMC12645228; doi:10.1111/1758-2229.70219)
Supplement: Supplementary file 1 — Figure S1: Comparison of growth characteristics of three strains of V. parahaemolyticus in SW and SSW. Figure S2: Comparison of growth characteristics of three strains of V. parahaemolyticus in TSB plus 1.5% NaCl. Figure S3: Expression levels of toxR (A), tlh (B), small RNA Spot42 (C) and RyhB (D) from V. parahaemolyticus S02, 2HP and 5HP under TSB condition at each time point. [file EMI4-17-e70219-s002.docx]

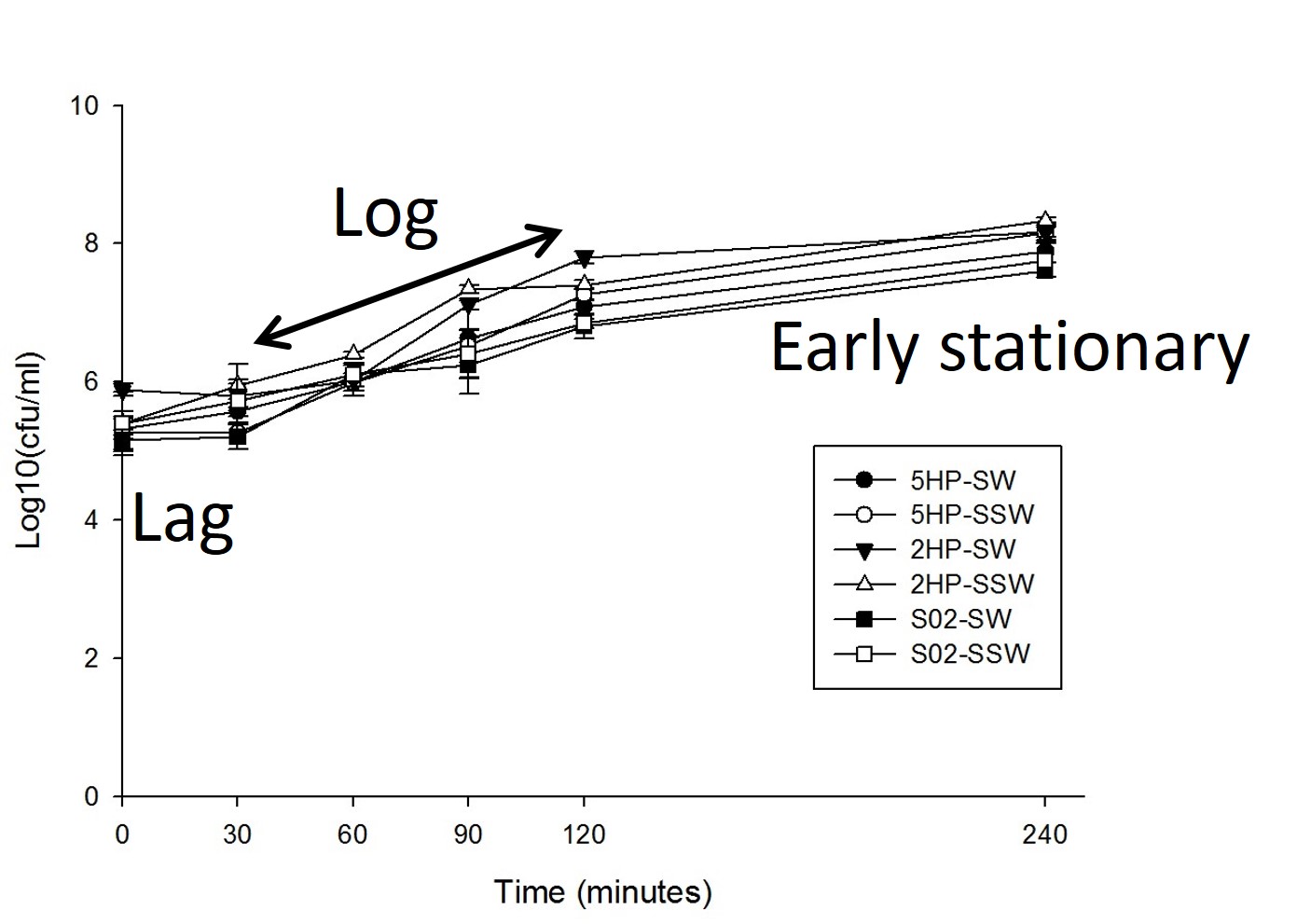


**Supplementary Figure S1** Comparison of growth characteristics of three strains of *V. parahaemolyticus* in SW and SSW. Circle, triangle, and square represent *V. parahaemolyticus* 5HP, 2HP, and S02, respectively. Closed and open symbols represent SW and SSW, respectively. Results are mean values and error bars represent standard deviation. (replicates = 3)


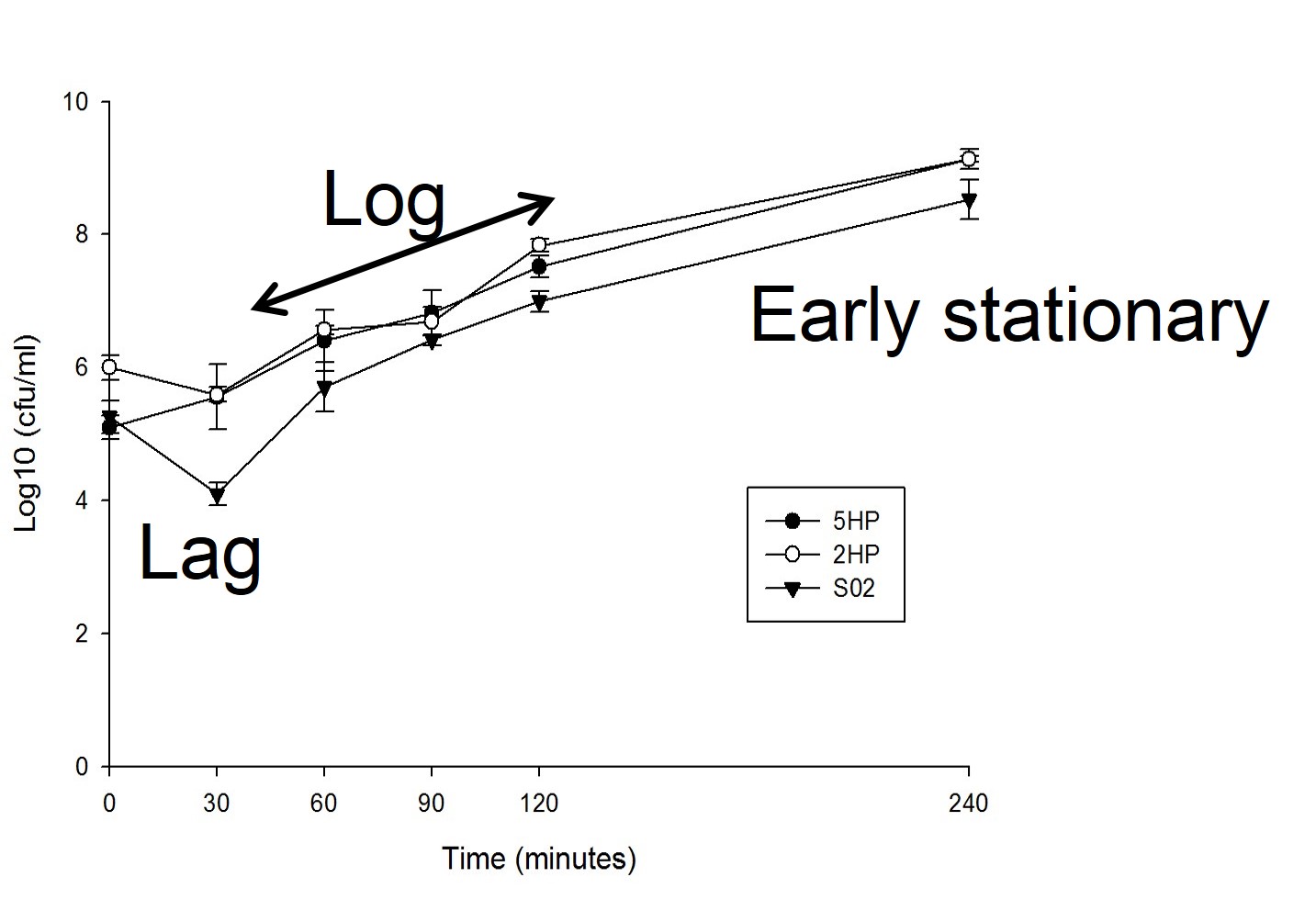


**Supplementary Figure S2** Comparison of growth characteristics of three strains of *V. parahaemolyticus* in TSB plus 1.5% NaCl. Closed circle, open circle, and closed inverted triangle represent *V. parahaemolyticus* 5HP, 2HP, and S02, respectively. Results are mean values and error bars represent standard deviation. (replicates = 3)


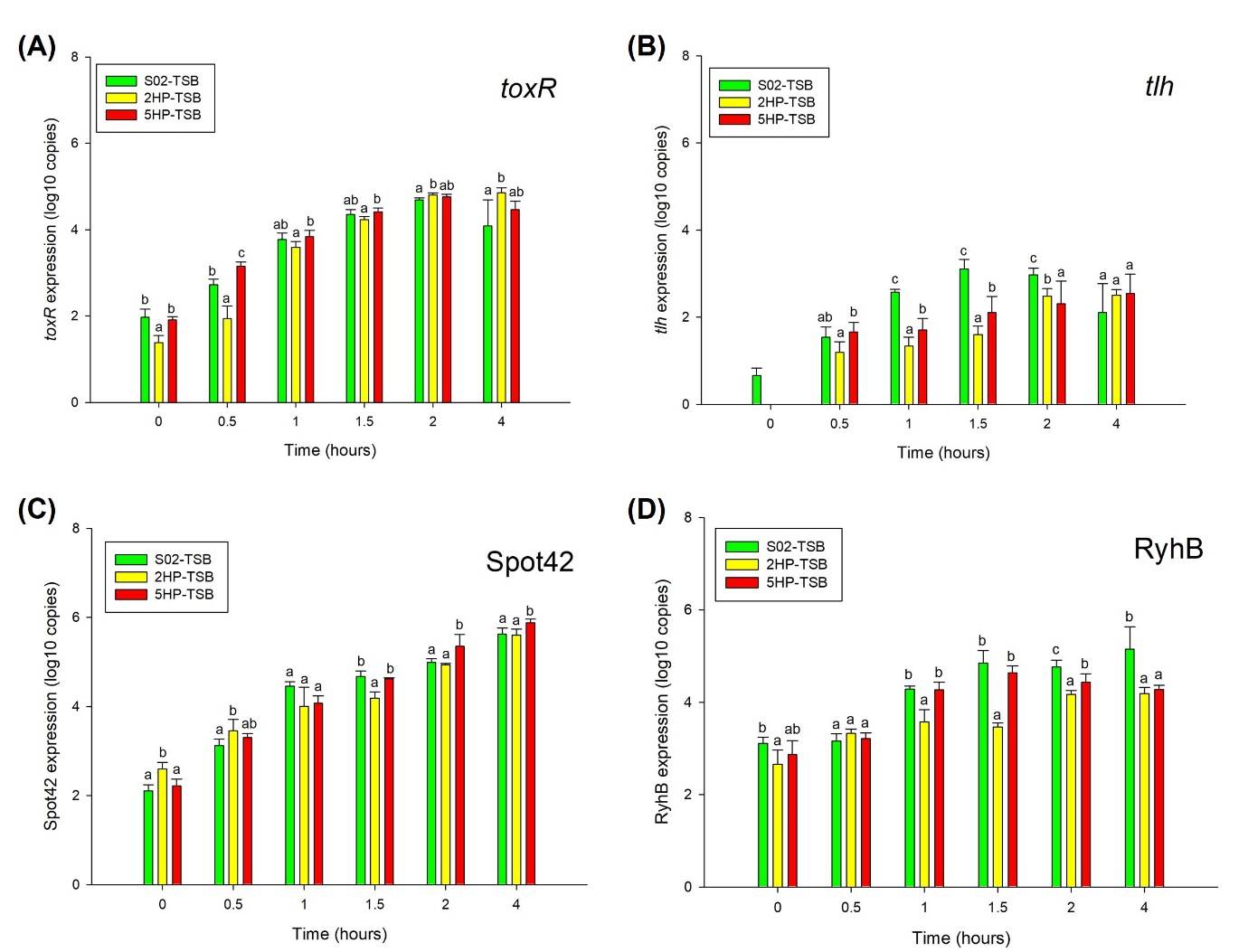


**Supplementary Figure S3** Expression levels of *toxR* (A), *tlh* (B), small RNA Spot42 (C), and RyhB (D) from *V. parahaemolyticus* S02, 2HP, and 5HP under TSB condition at each time point. Green, yellow, and red bars represent *V. parahaemolyticus* S02, 2HP, and 5HP, respectively. Different letters over error bars indicate statistically significant difference within time point. Results are mean values and error bars represent standard deviation in five independent experiments.
